# Clinical outcomes of abiraterone acetate and predictors of its treatment duration in metastatic castration‐resistant prostate cancer: Real‐world experience in the Southeast Asian cohort

**DOI:** 10.1002/cam4.3101

**Published:** 2020-05-06

**Authors:** Jasmine Lim, Akara Amantakul, Nisha Shariff, Bannakij Lojanapiwat, Adlinda Alip, Teng Aik Ong, Shankaran Thevarajah, Firdaus Ahmayuddin, Adeline Mathew, Supon Sriplakich, Jaraspong Vuthiwong, Flora Li Tze Chong, Marniza Saad

**Affiliations:** ^1^ Department of Surgery Faculty of Medicine University of Malaya Kuala Lumpur Malaysia; ^2^ Department of Surgery Faculty of Medicine Chiang Mai University Chiang Mai Thailand; ^3^ Department of Clinical Oncology Faculty of Medicine University of Malaya Kuala Lumpur Malaysia; ^4^ Department of Urology Queen Elizabeth Hospital Kota Kinabalu Malaysia; ^5^ Department of Radiotherapy and Oncology Sabah Women and Children Hospital Kota Kinabalu Malaysia

**Keywords:** chemotherapy, Malaysia, overall survival, progression‐free survival, PSA response, Thailand

## Abstract

It is of much interest to understand the efficacy of abiraterone acetate (AA) in routine clinical practice. We assessed the clinical outcome of AA in patients with metastatic castration‐resistant prostate cancer (mCRPC) and determined clinical factors associated with AA treatment duration in real‐world setting. This real‐world cohort consisted of 93 patients with mCRPC treated with AA in Thailand (58.1%) and Malaysia (41.9%). Primary endpoints were overall survival (OS) and biochemical progression‐free survival (bPFS). Secondary endpoints were predictors associated with AA treatment duration evaluated with Cox proportional hazards regression. Around 74% were chemotherapy‐naïve. The median AA treatment duration was 10 months (IQR 5.6‐17.1). Malaysians had a relatively lower median OS and bPFS (OS 17.8 months; 95% CI 6.4‐29.1, bPFS 10.4 months; 95% CI 8.8‐12.0) compared to Thais (OS 27.0 months; 95% CI 11.3‐42.7, bPFS 14.0 months; 95% CI 5.8‐22.2), although it did not achieve statistical significance (*P* > .05). Patients with longer AA treatment duration (>10 months) had lower risk of death and longer bPFS, compared to those with shorter AA treatment duration (≤10 months) (hazard ratio [HR] 0.10, 95% CI 0.05‐0.22 and HR 0.13, 95% CI 0.06‐0.25, respectively). Multivariable analysis showed that PSA at AA initiation, presence of PSA response and chemotherapy‐naive were independently associated with AA duration (*P* < .05). Abiraterone acetate is well‐tolerated in the Southeast Asian cohort with comparable survival benefits to other Asian populations in real‐world setting. Lower PSA levels at AA initiation, presence of PSA response, and chemotherapy‐naive were significant in determining AA treatment duration.

## Graphical abstract

Association of abiraterone acetate duration with (a) overall survival and (b) biochemical progress free survival in metastatic castration‐resistant prostate cancer.

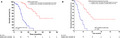

## 1 INTRODUCTION

Androgen deprivation therapy (ADT) has been the initial treatment of metastatic castration‐naïve prostate cancer since 1941.^1^ It comprises of either medical or surgical castration. Although 80% of patients are responsive to this treatment, most metastatic castration‐naïve patients develop metastatic castration‐resistant prostate cancer (mCRPC) within 12‐24 months after ADT initiation.^2^, ^3^ The addition of abiraterone or docetaxel to ADT has become current standard of care in metastatic castration‐naïve prostate cancer^4^; however, these combination of treatments were inaccessible to most patients in the real‐world clinical practice.^5^

The paradigm of mCRPC treatment is rapidly evolving with multitude of new treatments coming in post‐docetaxel from 2010. These treatments include novel androgen‐receptor‐targeting agent (abiraterone acetate, AA, and enzalutamide), radiopharmaceutical agent (radium‐223), chemotherapy (cabazitaxel), and immunotherapy (sipuleucel‐T and pembrolizumab).^4^ Abiraterone acetate (AA) is a selective, irreversible inhibitor of cytochrome‐P (CYP)‐17 enzyme that is critical for the production of androgens in the testes, adrenal glands, and prostate cancer cells. The efficacy of AA was established in large randomized controlled clinical trials showing significant survival benefits over placebo in both chemotherapy‐naïve and post‐chemotherapy patients.^6^, ^7^

However, its degree of clinical effectiveness may vary in the real‐world clinical practice.^8^, ^9^, ^10^, ^11^, ^12^, ^13^ In low‐middle income countries, most patients could not afford high‐cost drugs such as abiraterone (~US$ 2800/month).^5^ Therefore, identification of prognostic clinical markers is essential to aid clinicians in selecting patients who would most likely benefit from the AA treatment. In this study, we aimed to investigate the clinical outcome of patients with mCRPC undergoing AA and assess association of clinical characteristics with AA duration in the middle‐income Southeast Asian population.

## 2 MATERIALS AND METHODS

### 2.1 Study population

The study population consisted of 93 patients with mCRPC treated with AA; of which, 54 patients were from Chiang Mai University, Thailand while 39 patients were from three participating centers in Malaysia including University Malaya Medical Centre (n = 31), Queen Elizabeth Hospital (n = 5), and Sabah Women and Children Hospital (n = 3). All prostate cancer patients were diagnosed between 2002 and 2016 and started AA from March 2012 to October 2017. Patients were treated with 1000 mg AA once daily in combination with 5 mg prednisone twice a day. Serum prostate‐specific antigen (PSA), blood counts, liver, and renal profile were routinely tested for clinical and biochemical follow‐up during the treatment period. Regular imaging assessment was not mandatory unless clinical or biochemical progression was evident.

The study protocol was approved by the Regional Committee for Medical Research Ethics in Thailand and Malaysia. Informed consent was exempted by the medical research ethics committee because it was based on retrospective analyses of existing administrative and clinical data.

### 2.2 Procedures

Clinical and disease characteristics were retrieved from the hospital‐based patients’ case notes or electronic medical records. These data included age, comorbidities, PSA at diagnosis and baseline, Gleason score, Eastern Cooperative Oncology Group (ECOG) status, primary local treatment, primary ADT, and chemotherapy status. Primary ADT duration was defined as time from the date of first luteinizing hormone‐releasing hormone agonist/antagonist or orchidectomy to the start of AA. PSA doubling time was measured by determining the regression slope of the log PSA against time based on three consecutive PSA levels prior to AA initiation. PSA response was defined as ≥ 50% PSA decline from baseline within the first 12 weeks of therapy. Duration of AA treatment was determined from the time of first AA dose to treatment discontinuation owing to any reason including death from any causes, disease progression, or intolerable adverse events. Treatment‐related adverse events were graded according to the National Cancer Institute Common Terminology Criteria for Adverse Events (CTCAE) version 5.0.

### 2.3 Outcomes

The primary endpoints were overall survival (OS) and biochemical progression‐free survival (PFS), which were defined as time from first dose of AA to death, and to the first event of PSA progression or death, respectively. The definition of biochemical progressive disease was based on the Prostate Cancer Clinical Trials Working Group (PCWG‐3) criteria.^14^

### 2.4 Statistical analysis

The median OS and biochemical PFS comparison were estimated using the Kaplan‐Meier method. The primary statistical method of comparison for the time‐to‐event endpoints was log‐rank test stratified by country, chemotherapy status, and median duration of AA treatment. The Cox proportional hazards model was used to estimate the hazard ratio (HR) and its associated CI. Median variables, including PSA, ECOG status, Gleason score, duration of prior therapy, and time from ADT to mCRPC, were dichotomized at median or clinically meaningful cut‐off points. Multivariable analysis for AA treatment duration was performed to evaluate potential prognostic factors (ECOG status, PSA at AA initiation, PSA response, and chemotherapy status, *P* < .05; univariable analysis) with Cox proportional hazards regression. All statistical analysis was performed using SPSS for Windows version 21.0 (SPSS Inc). Two‐tailed *P* value < .05 was termed as statistically significant.

## 3 RESULTS

The median age at AA initiation was 70 years (interquartile range, IQR 65‐78) and 54.7% of patients were diagnosed with Gleason score ≥ 8 (Table 1). The proportion of chemo‐naïve and post‐chemo groups were 74.2% (69) and 25.8% (24), respectively (Table 1). Compared to Malaysians, Thai patients had a lower PSA level at diagnosis and comorbidity count as well as the use of primary local treatment and orchidectomy prior to AA therapy (*P* < .05; Table 1). No significant difference was found in all other variables between these two patient cohorts (Table 1).

**Table 1:** Baseline patient and disease characteristics

Characteristics	Frequency distribution, n (%), or median (IQR)^†^			*P* value
	Overall (N = 93)	Malaysia (n = 39)	Thailand (n = 54)	
*At initial diagnosis*				
Age (y)^†^	67 (61.5‐74)	66 (62‐69)	67.5 (60.8‐76)	.060
PSA (ng/mL)^†^	101 (30‐347)	223 (61.6‐1717)	76.7 (22.8‐238)	*.005*
*unknown	10	8	2	
Gleason score				
≤6	15 (17.4)	6 (18.2)	9 (17.0)	.098
7	24 (27.9)	8 (24.2)	16 (30.2)	
≥8	47 (54.7)	19 (57.6)	28 (52.8)	
*unknown	7	6	1	
Presence of metastases (M1) at initial diagnosis of prostate cancer				
No	22 (24.7)	9 (25.7)	13 (24.1)	.861
Yes	67 (75.3)	26 (74.3)	41 (75.9)	
*unknown	4	4	0	
*Prior therapy*				
Primary local treatment				
Radical prostatectomy	4 (4.3)	0	4 (7.4)	*<.001*
Radiotherapy	11 (11.8)	10 (25.6)	1 (1.9)	
None	78 (83.9)	29 (74.4)	49 (90.7)	
Primary ADT				
LHRH agonist	56 (60.2)	27 (69.2)	29 (53.7)	*.017*
LHRH antagonist	9 (30.1)	0	9 (29.6)	
Orchidectomy	28 (9.7)	12 (30.8)	16 (16.7)	
Duration of primary ADT	30.3 (15.3‐55.9)	31.4 (14.6‐61.7)	28.9 (15.4‐51.4)	.559
*unknown	3	3	0	
PSA doubling time (mo)^†^	2.4 (1.5‐4.2)	2 (1.4‐3.1)	2.4 (1.5‐5.1)	.417
*unknown	4	2	2	
Time from ADT to mCRPC (mo)^†^	21 (9.5‐47.5)	21.1 (9‐48.5)	19.8 (11.4‐47.4)	.762
*unknown	4	4	0	
Chemotherapy status				
Chemo‐naïve	69 (74.2)	28 (71.8)	41 (75.9)	.653
Post‐chemo	24 (25.8)	11 (28.2)	13 (24.1)	
*At abiraterone initiation*				
Age (y)^†^	70 (65‐78)	68 (65‐73)	73 (65.8‐81)	.115
PSA (ng/mL)^†^	66.6 (20.6‐165.5)	82.4 (33‐223)	47.5 (12.5‐187.5)	.071
*unknown	2	0	2	
ECOG status				
0‐1	68 (76.4)	29 (76.3)	39 (76.5)	.059
2	15 (16.9)	4 (10.5)	11 (21.5)	
3‐4	6 (6.7)	5 (13.2)	1 (2.0)	
*unknown	4	1	3	
Comorbidity count				
0	36 (38.7)	8 (20.5)	28 (51.9)	*.012*
1	26 (28.0)	14 (35.9)	12 (22.2)	
2	21 (22.5)	10 (25.7)	11 (20.3)	
≥3	10 (10.8)	7(17.9)	3 (5.6)	
Year of initiation				
2012‐2014	17 (18.3)	11 (28.2)	6 (11.1)	*.027*
2015	27 (29.0)	10 (25.6)	17 (31.5)	
2016	28 (30.1)	14 (35.9)	14 (25.9)	
2017	21 (22.6)	4 (10.3)	17 (31.5)	

Abbreviations: ADT, androgen deprivation therapy; ECOG, Eastern Cooperative Oncology Group; IQR, interquartile range; mCRPC, metastatic castrate‐resistant prostate cancer; PSA, prostate‐specific antigen.

† Indicates that the value of these factors were stated in median (IQR).

Colour shaded values indicates statistical significant *P*‐values.

The median duration of AA treatment was 10 months (IQR 5.6‐17.1) at the time of last follow‐up. Around 60% of patients achieved ≥ 50% of PSA decline from baseline within 12 weeks. Reasons for treatment discontinuation were disease progression (80.6%; 29/36), adverse events (11.1%; 4/36), and financial problems (8.3%; 3/36). The most common grade 3‐4 adverse events were fatigue (5/93; 5.4%) and electrolytes abnormalities (3/93; 3.2%).

**Figure 1:**
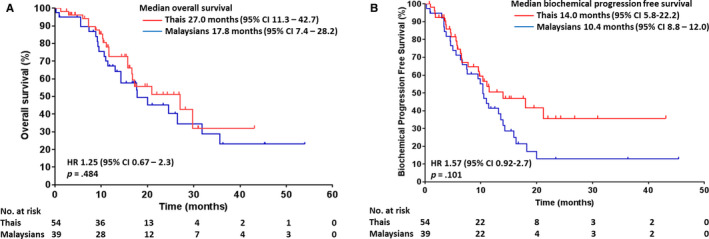
A, Overall survival and B, biochemical progression‐free survival for Malaysian and Thai patients with mCRPC treated with abiraterone acetate

**Figure 2:**
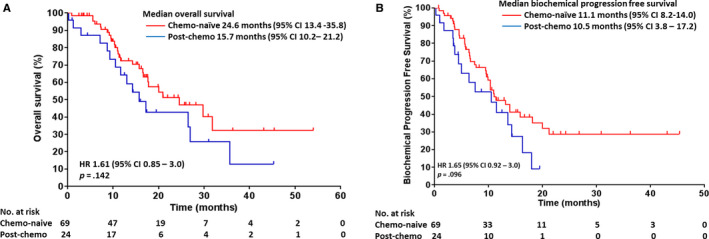
A, Overall survival and B, biochemical progression‐free survival for chemo‐naïve and post‐chemo patients with mCRPC treated with abiraterone acetate

The median OS of Malaysians was lower (17.8 months; 95% CI 6.4‐29.1) than Thais (OS 27.0 months; 95% CI 11.3‐42.7), although it did not achieve statistical significance (*P* = .484) (Figure 1A). A comparable median biochemical PFS was observed in both Malaysian (10.4 months; 95% CI 8.8‐12.0) and Thai cohorts (14.0 months; 95% CI 5.8‐22.2) (Figure 1B). There were no significant difference in median OS and biochemical PFS between chemotherapy‐naïve and post‐chemotherapy patients with mCRPC (Figure 2A,B).

**Figure 3:**
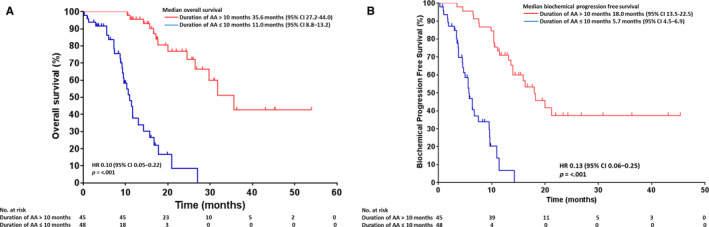
Association of abiraterone acetate duration with A, overall survival and B, biochemical progression‐free survival

In addition, the risk of death and biochemical progression were significantly decreased in patients with longer duration of AA (>10 months) compared to those of shorter duration of AA (≤10 months) (hazard ratio [HR] 0.10, 95% CI 0.05‐0.22 and HR 0.13, 95% CI 0.06‐0.25, respectively, *P* < .001; Figure 3). For instance, median OS was 35.6 months (95% CI 27.2‐44.0) in patients with > 10 months AA therapy and 11.0 months (8.8‐13.2) in those with duration of AA ≤ 10 months (Figure 3A).

Further analysis revealed that longer duration of AA was associated with ECOG status, PSA at AA initiation, chemotherapy status, and PSA response (*P < *.05) in the univariable analysis (Table 2). The multivariable‐adjusted model showed that high PSA at AA initiation (>61 ng/mL) (HR 2.04, 95% CI 1.10‐3.79), the presence of PSA response (HR 0.42, 95% CI 0.22‐0.80), and prior exposure to chemotherapy (HR 1.91, 95% CI 1.03‐3.54) remained significant determinants of duration of AA treatment (Table 3).

**Table 2:** Comparison of factors associated with duration of abiraterone treatment

Factors	Median (mo), IQR	Events/ N	HR (95%CI)	*P* value
	Duration of AA	Discontinuation of AA		
Patients cohort				
Malaysia	11.8 (5.9‐17.4)	25/39	1.00	
Thailand	9.7 (4.6‐15.6)	25/54	0.87 (0.50‐1.53)	.630
Age at AA initiation (y)	10 (5.6‐17.05)	50/93	1.00 (0.97‐1.03)	.875
Gleason score				
≤7	11.4 (5.6‐19.8)	17/39	1.00	
>7	9.6 (5.4‐14.3)	29/47	1.78 (0.97‐3.25)	.063
ECOG status				
≤1	11.5 (5.8‐17.4)	33/68	1.00	
>1	8.8 (5.2‐13.1)	15/21	1.94 (1.05‐3.60)	.035
Comorbidity count				
≤1	9.7 (5.8‐16.7)	32/62	1.00	
>2	10.0 (4.4‐17.7)	18/31	1.05 (0.59‐1.88)	.868
Duration of primary ADT				
≤30.3 mo	8.3 (4.2‐14.2)	25/45	1.00	
>30.3 mo	12.9 (6.6‐17.7)	24/45	0.73 (0.41‐1.27)	.263
Time from ADT to mCRPC				
≤21 mo	7.9 (4.6‐17.2)	24/45	1.00	
>21 mo	12.0 (6.6‐17.0.0)	25/44	0.98 (0.56‐1.74)	.950
PSA doubling time				
≤2.4 mo	10.2 (4.7‐17.3)	26/48	1.00	
>2.4 mo	9.8 (5.8‐17.2)	21/41	0.95 (0.53‐1.69)	.857
PSA at AA initiation				
≤61 ng/mL	12.5 (5.7‐17.5)	16/45	1.00	
>61 ng/mL	9.5 (5.3‐16.9)	33/46	2.31 (1.27‐4.20)	.006
PSA response*				
No	6.2 (3.5‐10.0)	20/34	1.00	
Yes	13.6 (8.3‐22.0)	24/47	0.43 (0.23‐0.79)	.006
Chemotherapy status				
Chemo‐naïve	10.6 (5.7‐17.2)	33/69	1.00	
Post‐chemo	7.9 (3.8‐14.2)	17/24	1.80 (1.00‐3.24)	.050

Abbreviations: AA, abiraterone acetate; ADT, androgen deprivation therapy; CI, confidence interval; ECOG, Eastern Cooperative Oncology Group; HR, hazard ratio; IQR, interquartile range; mCRPC, metastatic castrate‐resistant prostate cancer; PSA, prostate‐specific antigen.

^*^ ≥50% PSA decline from baseline within 12 wks.

Colour shaded values indicates statistical significant *P*‐values.

**Table 3:** Multivariable analysis of factors associated with duration of abiraterone treatment

Factors	Hazard ratio (95%CI)	*P* value
ECOG status		
≤1	1.00	
>1	1.75 (0.96‐3.44)	.068
PSA at AA initiation (ng/mL)		
≤61	1.00	
>61	2.04 (1.10‐3.79)	.024
PSA response*		
No	1.00	
Yes	0.42 (0.22‐0.80)	.008
Chemotherapy status		
Chemo‐naïve	1.00	
Post‐chemo	1.91 (1.03‐3.54)	.040

Abbreviations: AA, abiraterone acetate; CI, confidence interval; ECOG, Eastern Cooperative Oncology Group.

* ≥50% PSA decline from baseline within 12 wks.

Colour shaded values indicates statistical significant *P*‐values.

## 4 DISCUSSION

Findings from this multicenter study provide an insight into the efficacy and toxicity of AA in patients with mCRPC in the real‐world setting. We demonstrated that initial PSA level, chemotherapy status and PSA response were idependent factors associated with duration of AA treatment.

The clinical efficacy including OS, PFS, and PSA response in our chemotherapy‐naïve mCRPC group was similar with other Asia countries including Japan,^9^ Hong Kong,^8^ China,^15^ and Singapore^16^ (Table 4). The median AA treatment duration of our cohort (10 months) was similar to other real‐world studies,^8^, ^17^, ^18^ although slightly shorter than those reported in COU‐AA‐302 trial.^7^ Although proportion of PSA response (PSA decline ≥ 50% within 12 weeks) were comparable with COU‐AA‐302 trial,^7^ a lower median OS was observed in our cohort (Table 4). We hypothesize that the inferior survival outcome of our chemotherapy‐naïve patients may be attributed to higher proportion of de‐novo metastatic disease and a relatively high‐disease burden in our patient cohorts. A higher median baseline PSA level (66.6 ng/mL) was found in our cohort than COU‐AA‐302 study (42 ng/mL).^7^ The presence of ECOG ≥ 2 (21.2%) and visceral metastasis (15.5%) in our chemotherapy‐naïve patients may also account for unsatisfactory survival results as those were exclusion criteria in the COU‐AA‐302 trial.^7^

**Table 4:** Clinical outcome of chemotherapy‐naïve patients with mCRPC in the present study^†^, pivotal clinical trial, and Asian cohorts

Factors	COU‐AA‐302^5^ (n = 546)	Japan^9^ (n = 113)	Hong Kong^8^ (n = 58)	China^15^ (n = 103)	Singapore^16^ (n = 163)	Malaysia^†^ (n = 28)	Thailand^†^ (n = 41)
Median time on AA (range)	13.8 (0.3‐34.9)	n.a	6.8 (0.6‐21.5)	n.a	n.a	11.8 (0.1‐61.0)	9.7 (0.8‐43.1)
Median OS (95% CI)	34.7 (32.7‐36.8)	n.a	18.1 (9.9‐25.0)	27.0 (n.a)	20.0 (18.3‐22.9)	17.8 (7.4‐28.2)	27 (11.3‐42.7)
Median PFS (95% CI)	16.5^a^	9^b^	6.7^c^ (4.5‐14.7)	14.0^b^ (n.a)	9.6^b^ 7.8‐11.7)	10.4^b^ (8.8‐12.0)	14^b^ (5.8‐22.2)
PSA decline ≥ 50% within 12 wks, n(%)	374 (58)	60 (53.1)	36 (62.1)	56 (54.4)	n.a	13 (56.5)	22 (57.9)
Grade ≥ 3 adverse events, n (%)	290 (54)	5 (4.4)	9 (15.5)	n.a	n.a^d^	6 (21.4)	9 (22)

Abbreviations: AA, abiraterone acetate; CI, confidence interval; IQR, interquartile range; n.a, not available; OS, overall survival; PFS, progression‐free survival.

† Indicates that the Malaysia and Thailand cohorts were from the present study.

^a^ Radiological PFS.

^b^ Biochemical PFS.

^c^ Combination of clinical, radiological, or biochemical PFS.

^d^ Toxicity data for chemotherapy‐naïve patients alone were unavailable.

Comparing to the COU‐AA‐302 trial, the rate of grade ≥ 3 adverse events was reported between 15.5% and 22.0% in the real‐world Asian cohorts including Hong Kong^8^ and present study (Table 4). Interestingly, only 4.4% of Japanese treated with AA exhibited grade ≥ 3 adverse events.^9^ This is in line with earlier findings showing anticancer drugs including those against mCRPC were more tolerable in Asians particularly Japanese patients than Western population.^19^, ^20^ It is worth noting that variations found in the findings of retrospective observational studies may attribute to different study design, duration of follow‐up, and patient assessment.

We demonstrated that a longer AA treatment duration (>10 months) was predictive of favorable OS and biochemical PFS in patients with AA in this study. These findings suggest that short response to AA may be associated with primary resistance to AR‐pathway targeted therapy, leading to poorer survival benefits.^21^ Multivariable analysis further uncovered that prior exposure to chemotherapy and the absence of PSA response were associated with a shorter duration of AA. Based on results from COU‐AA‐301 and COU‐AA‐302, the PSA response rate (⩾50% decline in PSA from baseline) was 62% among chemotherapy‐naïve patients with better PFS of 16.5 months compared to post‐chemotherapy group having 29% of PSA response and 5.6 months of PFS.^6^, ^7^ These results may suggest modest activity of docetaxel following abiraterone owing to some degrees of cross‐resistance between chemotherapy and abiraterone.^22^, ^23^ Nevertheless, men undergoing prior chemotherapy may have greater tumor burden, which attributes to more diverse AR‐independent signaling pathways.^24^

Heterogeneity of responses to AA in mCRPC highlights the importance of biomarkers development for personalized medicine. Recent findings revealed that detection of AR splice variant 7 messenger RNA (AR‐V7) in circulating tumor cells was associated with increased risk of primary resistance to abiraterone and enzalutamide in CRPC patients.^25^ Galeterone, a novel potent anti‐androgen inducing CYP17 lyase inhibitor, AR degradation and antagonism, is currently under the pivotal phase III biomarker selection design trial targeting AR‐V7–positive patients.^26^ Additionally, DNA‐repair pathway has emerged as potential targets for genetically targeted treatment in mCRPC. Olaparib, a polyADP‐ribose polymerase (PARP) inhibitor, is currently used for treatment of ovarian cancer with BRCA2 loss. In a phase II open‐label single‐arm study, 88% of patients with mCRPC with DNA‐repair gene aberrations including those of *BRCA2* loss responded to olaparib treatment with significant improved PFS and OS compared to mutation‐negative group.^27^ Despite the evidence of clinical benefit of new drugs, cost remains a major determinant of global access to prostate cancer treatment particularly in low‐ and middle‐income countries.^5^

There are limitations in this multicenter study. First, comparative analyses were not conducted between pre‐chemotherapy and post‐chemotherapy groups, owing to the limited sample size. Second, we prefer the term “duration of treatment” to actual “response duration” because it reflects “real‐world” routine clinical practice. Physicians exercise clinical judgment in discontinuing treatments deemed medically futile. Third, regular imaging was not mandatory unless patients presented with clinical or biochemical progression. This may deprive the chances of some patients receiving other life‐prolonging treatment earlier upon the presence of radiological progression. Finally, this retrospective design is subject to incomplete data collection or variable assessments, under‐reporting of adverse events, and potential selection bias.

In summary, AA was well‐tolerated in our cohort with comparable OS and PFS to other Asian populations in real‐world setting. The median OS was much shorter compared to pivotal clinical trials. We confirm that lower PSA levels at AA initiation, presence of PSA response, and no prior chemotherapy could serve as prognostic markers to determine optimal duration of AA treatment. Access to life‐prolonging therapy in a sequential manner may achieve optimal prolongation of survival.

## CONFLICT OF INTEREST

All authors have nothing to disclose.

## AUTHOR CONTRIBUTIONS

Conceived and designed the study: JL, NS, AA, and MS. Performed the study: JL, AA, NS, BL, AA, TAO, ST, FA, AM, SS, JV, FLTC, and MS. Analyzed the data: JL, AA, NS, AA, and MS. Contributed materials/analysis tools: JL, AA, NS, BL, AA, TAO, ST, FA, AM, SS, JV, FLTC, and MS. Contributed to the writing of the manuscript: JL, BL, TAO, AA, and MS.

## Data Availability

The data used to support the findings of this study are available from the corresponding author upon request.

## References

[1] Huggins C , Hodges CV . Studies on prostatic cancer. I. The effect of castration, of estrogen and androgen injection on serum phosphatases in metastatic carcinoma of the prostate. Cancer Res. 1941;1:293‐297. 10.3322/canjclin.22.4.232 4625049

[2] Mostaghel EA , Montgomery B , Nelson PS . Castration‐resistant prostate cancer: targeting androgen metabolic pathways in recurrent disease. Urol Oncol. 2009;27:251‐257. 19414113 10.1016/j.urolonc.2009.03.016 PMC2705999

[3] Sweeney CJ , Chen Y‐H , Carducci M , et al. Chemohormonal therapy in metastatic hormone‐sensitive prostate cancer. New Engl J Med. 2015;373:737‐746. 26244877 10.1056/NEJMoa1503747 PMC4562797

[4] NCCN clinical practice guidelines in oncology. Prostate Cancer. Version 2, 2019. 10.6004/jnccn.2019.0023 31085757

[5] Saad M , Alip A , Lim J , et al. Management of advanced prostate cancer in a middle‐income country: real‐world consideration of the Advanced Prostate Cancer Consensus Conference 2017. BJU Int. 2019;124:373‐382. 31077523 10.1111/bju.14807 PMC6851975

[6] Fizazi K , Scher HI , Molina A , et al. Abiraterone acetate for treatment of metastatic castration‐resistant prostate cancer: final overall survival analysis of the COU‐AA‐301 randomised, double‐blind, placebo‐controlled phase 3 study. Lancet Oncol. 2012;13:983‐992. 22995653 10.1016/S1470-2045(12)70379-0

[7] Ryan CJ , Smith MR , Fizazi K , et al. Abiraterone acetate plus prednisone versus placebo plus prednisone in chemotherapy‐naive men with metastatic castration‐resistant prostate cancer (COU‐AA‐302): final overall survival analysis of a randomised, double‐blind, placebo‐controlled phase 3 study. Lancet Oncol. 2015;16:152‐160. 25601341 10.1016/S1470-2045(14)71205-7

[8] Poon DM , Chan K , Lee SH , et al. Abiraterone acetate in metastatic castration‐resistant prostate cancer ‐ the unanticipated real‐world clinical experience. BMC Urol. 2016;16:12. 27001043 10.1186/s12894-016-0132-z PMC4802641

[9] Miyake H , Hara T , Terakawa T , Ozono S , Fujisawa M . Comparative assessment of clinical outcomes between abiraterone acetate and enzalutamide in patients with docetaxel‐naive metastatic castration‐resistant prostate cancer: experience in real‐world clinical practice in Japan. Clin Genitourin Cancer. 2017;15:313‐319. 27424256 10.1016/j.clgc.2016.06.010

[10] Rocha J , Aprikian AG , Vanhuyse M , et al. Impact of abiraterone acetate with and without prior docetaxel chemotherapy on the survival of patients with metastatic castration‐resistant prostate cancer: a population‐based study. CMAJ Open. 2017;5:E265‐E272. 10.9778/cmajo.20160082 PMC5378527 28401143

[11] Rescigno P , Lorente D , Bianchini D , et al. Prostate‐specific antigen decline after 4 weeks of treatment with abiraterone acetate and overall survival in patients with metastatic castration‐resistant prostate cancer. Eur Urol. 2016;70:724‐731. 26965561 10.1016/j.eururo.2016.02.055

[12] Thortzen A , Thim S , Roder MA , Brasso K . A single‐center experience with abiraterone as treatment for metastatic castration‐resistant prostate cancer. Urol Oncol. 2016;34(291):e1‐7. 10.1016/j.urolonc.2016.02.013 26971191

[13] Cindolo L , Natoli C , De Nunzio C , et al. Safety and efficacy of abiraterone acetate in chemotherapy‐naive patients with metastatic castration‐resistant prostate cancer: an Italian multicenter "real life" study. BMC Cancer. 2017;17:753. 29126389 10.1186/s12885-017-3755-x PMC5681753

[14] Scher HI , Morris MJ , Stadler WM , et al. Trial design and objectives for castration‐resistant prostate cancer: updated recommendations from the prostate cancer clinical trials working group 3. J Clin Oncol. 2016;34:1402‐1418. 26903579 10.1200/JCO.2015.64.2702 PMC4872347

[15] Lin GW , Li GX , Dai B , et al. Clinical activity of abiraterone plus prednisone in docetaxel‐naomicronve and docetaxel‐resistant Chinese patients with metastatic castration‐resistant prostate cancer. Asian J Androl. 2019;21:131‐136. 30560837 10.4103/aja.aja_85_18 PMC6413557

[16] Chan J , Yap SY , Fong YC , et al. Real‐world outcome with abiraterone acetate plus prednisone in Asian men with metastatic castrate‐resistant prostate cancer: the Singapore experience. Asia Pac J Clin Oncol. 2020;16:75‐79. 31713349 10.1111/ajco.13241

[17] Boegemann M , Khaksar S , Bera G , et al. Abiraterone acetate plus prednisone for the Management of Metastatic Castration‐Resistant Prostate Cancer (mCRPC) without prior use of chemotherapy: report from a large, international, real‐world retrospective cohort study. BMC Cancer. 2019;19:60. 30642291 10.1186/s12885-019-5280-6 PMC6332550

[18] McKay RR , Werner L , Fiorillo M , Nakabayashi M , Kantoff PW , Taplin ME . Predictors of duration of abiraterone acetate in men with castration‐resistant prostate cancer. Prostate Cancer Prostatic Dis. 2016;19:398‐405. 27502737 10.1038/pcan.2016.31 PMC6034654

[19] Matsubara N , Uemura H , Satoh T , et al. A phase 2 trial of abiraterone acetate in Japanese men with metastatic castration‐resistant prostate cancer and without prior chemotherapy (JPN‐201 study). Jpn J Clin Oncol. 2014;44:1216‐1226. 25320340 10.1093/jjco/hyu149 PMC4243579

[20] Satoh T , Uemura H , Tanabe K , et al. A phase 2 study of abiraterone acetate in Japanese men with metastatic castration‐resistant prostate cancer who had received docetaxel‐based chemotherapy. Jpn J Clin Oncol. 2014;44:1206‐1215. 25425730 10.1093/jjco/hyu148 PMC4243578

[21] Fitzpatrick JM , Bellmunt J , Fizazi K , et al. Optimal management of metastatic castration‐resistant prostate cancer: highlights from a European Expert Consensus Panel. Eur J Cancer. 2014;50:1617‐1627. 24703899 10.1016/j.ejca.2014.03.010

[22] Mezynski J , Pezaro C , Bianchini D , et al. Antitumour activity of docetaxel following treatment with the CYP17A1 inhibitor abiraterone: clinical evidence for cross‐resistance? Ann Oncol. 2012;23:2943‐2947. 22771826 10.1093/annonc/mds119

[23] Schweizer MT , Zhou XC , Wang H , et al. The influence of prior abiraterone treatment on the clinical activity of docetaxel in men with metastatic castration‐resistant prostate cancer. Eur Urol. 2014;66:646‐652. 24491307 10.1016/j.eururo.2014.01.018 PMC4110192

[24] Bluemn EG , Coleman IM , Lucas JM , et al. Androgen receptor pathway‐independent prostate cancer is sustained through FGF signaling. Cancer Cell. 2017;32(474–89):e6. 10.1016/j.ccell.2017.09.003 PMC5750052 29017058

[25] Antonarakis ES , Lu C , Wang H , et al. AR‐V7 and resistance to enzalutamide and abiraterone in prostate cancer. New Engl J Med. 2014;371:1028‐1038. 25184630 10.1056/NEJMoa1315815 PMC4201502

[26] Njar VC , Brodie AM . Discovery and development of Galeterone (TOK‐001 or VN/124‐1) for the treatment of all stages of prostate cancer. J Med Chem. 2015;58:2077‐2087. 25591066 10.1021/jm501239f

[27] Mateo J , Carreira S , Sandhu S , et al. DNA‐repair defects and olaparib in metastatic prostate cancer. New Engl J Med. 2015;373:1697‐1708. 26510020 10.1056/NEJMoa1506859 PMC5228595

